# Cerebro-Spinal Meningitis, or Spotted Fever

**Published:** 1874-05

**Authors:** James S. Whitmire

**Affiliations:** Metamora, Ill.


A MONTHLY RECORD OF

*Medicine, Surgery and the Collateral Sciences.*

EDITED BY J. ADAMS ALLEN, M.D., LL.D.; AND WALTER HAY, M.D.

VOL. XXXI.—MAY, 1874.—No. 5.

### Original Communications.

ARTICLE I.—*Cerebro-Spinal Meningitis, or, Spotted Fever.* Read before the Joint Session of the Woodford and Marshall County Medical Societies, assembled at Winona, Ill., January 6th, A. D. 1874. By JAMES S. WHITMIRE, M.D., of Metamora, Ill.

MESSRS. PRESIDENT AND GENTLEMEN OF THIS JOINT ASSOCIATION:

At the last meeting of the Woodford County Medical Society, held at Eureka, I was designated by our President to make a special report at the joint meeting of these Societies on the subject of Cerebro-Spinal Meningitis, so-called, as it appeared in Woodford county during the latter part of the winter and spring of 1872, and the latter part of the fall and winter of 1872–73. In accordance, therefore, with such appointment, I would beg leave to submit the following report.

This disease made its first appearance in the vicinity of Metamora and Spring Bay simultaneously, about the middle of February, and from these places it spread east and north respectively, for many miles; but within this range there were certain localities that suffered more severely than others. In the two places particularly mentioned, and in their vicinity, it assumed all the malignancy and produced all the horrors of a terrible and alarming plague. It was clearly epidemic in our range of practice, and though other portions of the county suffered to some extent, yet in those it seemed to appear more in a sporadic form, there occurring no more than one, two or three cases in a neighborhood, though, even in that case, it seemed to abate none of its malignancy; neither, in any locality, did it seem to be any respecter of persons, because it attacked individuals of all ages, sexes and conditions, from sixty years down to the infant at the mother's breast.

The disease again made its appearance in the vicinity of Spring Bay during the latter part of November of the same year, assuming, from the commencement, an epidemic character, and did not abate, either its spread, or its violence, till February, 1873. To speak more particularly of our spring epidemic of spotted fever, it became so alarmingly prevalent during the month of April, and the mortality was so great, that the whole population became greatly agitated and alarmed; and the fear was so general, that for a time it was almost impossible to procure aid for taking care of the sick, lest there should be either contagion or infection connected with it. During this time, the late Judge Richmond was holding court in Metamora, and the alarm was so general on account of the prevalence and fatality of the disease, that a panic was produced among all classes, so that it was with the greatest difficulty the court, with all its judicial authority, could keep the witnesses and empaneled jurors from making an immediate simultaneous stampede.

The spring epidemic at Spring Bay was neither so wide-spread nor fatal as it was with us in the vicinity of Metamora and east, but it extended up and down the Illinois river bottom for eight or ten miles, and back into the country among the bluffs for several miles from the river, but it followed, principally, the creeks and swales that carried the debris from the hill country on its way to the Illinois river. I have frequently witnessed sporadic cases of this disease in the autumn of different seasons since 1848, but I never before witnessed the disease assume an epidemic character, at this season of the year, till it made its appearance in that manner in November, 1872, when it put in an appearance at Spring

Bay and along the river bottom, in its most deadly character, and continued till the early spring of 1873.

In the spring epidemic, along the Illinois river, the disease seemed to be, and was, quite amenable to the influence of medicine, and my friend, Dr. J. G. Zeller, who lives at the Bay, had all who were attacked in the range of his practice to attend. He was much more successful in his treatment, at this time, than we were who practiced on the prairies, nearly all, or at least a large proportion, of his patients making a speedy recovery. He did not, during the spring epidemic, lose to exceed fifteen per cent. of his patients, so that reckoning from his own standpoint of success, he very naturally came to the conclusion that the disease was but little, if any, more fatal than others of our endemic diseases, and that its fatality with us on the prairies away from the river, was owing to bad management or other remediable causes. In his cases the most alarming symptoms usually disappeared on the administration of quinine, but still there remained after its administration the persistent contraction of the posterior cervical and the dorsal muscles, but by the continuation of tonic doses of this drug the contraction gradually gave way, and his patients were soon traveling on the highway to health and happiness. But the fact that the disease yielded so certainly to the influence of quinine, and that its characteristic symptoms slowly relaxed under its continuance, makes the presumption strong, notwithstanding most of the distinguishing features of so-called cerebro-spinal meningitis were present—and this, no doubt, on account of the epidemic cause of the disease being present—that miasmata was the principal factor in the disease as it prevailed at this time.

But the epidemic that occurred the following fall and winter presented itself with the same virulence that had attended its visitation during the spring previously, in the vicinity of Metamora. One family living in Spring Bay, during the epidemic lost three children, between the ages of six and fourteen years, with the disease in less than three weeks, two of whom were attacked and died within the same twenty-four hours, and buried in the same grave. I had the honor of being called in consultation with Dr. Zeller in three cases, one of which was his own child; and while on this visit, the Doctor was pleased to take me with him to see seven or eight others who had been stricken with the same disease, all lying within a radius of half a mile; and of that number there was but one perfect recovery; two got up; one of them was perfectly blind, and the other permanently deaf. In this epidemic, I think that I am safe in saying that there were fully fifty per cent. of fatal cases, probably more, and some of the cases lingered for over one hundred days before they died, and one perfectly recovered when convalescence was not fully established till after that period.

In our spring epidemic on the table lands, we had very much such a time and experience as that of Dr. Zeller during the fall and winter, and the disease assumed the same malignant type. One case that came under my care was a young lady, Miss W., aged sixteen years, in whom the disease was presented in its most deadly form. She arose from her bed in the morning, made a fire, and proceeded to get breakfast for the family; after breakfast her father and mother went to Washington, four miles distant, to do some shopping. On their return at 12 o'clock, M., they found her lying on the bed in a partially insensible condition; alarmed at this, I was immediately sent for, and arrived about 2 o'clock, P. M. The surface of my patient was cool and pallid; the pupils of both eyes were dilated to their utmost extent; her pulse was beating at the rate of only sixty per minute, moderately full, but soft and feeble; her hearing was obtuse, and she answered questions only in monosyllables, and not then and in that manner unless she was sharply addressed; besides, her comprehension was exceedingly dull. I ordered her one tablespoonful of whisky, in hot milk, every five, ten, or fifteen minutes, and directed that she should be packed in a blanket wrung out of hot water, but before we could get the water hot she was a corpse.

My friend, Dr. A. H. Kinnear, I believe, had the misfortune to be called to one or two similar cases, where the patients succumbed to the toxic effects of the cause before there was any chance for medication. I simply mention these cases in connection with those of Dr. Zeller, that died so suddenly, because I may have occasion to refer to them again in this paper when I come to treat of the etiology of the disease.

It is not my purpose, in this paper, to enter the field of history in regard to this disease, as it has made its appearance in this and other countries at different times; neither is it my purpose to give the general or diagnostic signs of the disease, because these are sufficiently detailed in our standard works, and have now become a part and parcel of our reliable medical literature, about which there is no controversy. But my purpose will be to direct the mind of the inquirer to the cause or causes that may or may not combine to produce this disease; and these we can only approximate by observation and comparison, always keeping in mind the condition and circumstances under which it is most generally manifested, and in so doing, we will scarcely lose sight of the mooted question, whether this is a zymotic or other form of disease. In this connection I will state, as my opinion, that this disease originates from the direct influence of a blood poison, which is generated by extraneous causes, and is, most probably, received into the blood through the instrumentality of respiration; that this is an extrinsic, impalpable poison, and that it is cumulative in its nature, rather than a reproducer of itself—the latter property belonging only to the contagious zymoma. Or, perhaps it would be better to make the statement that the animal economy, through the blood, is capable of receiving and retaining a very considerable amount of the poison for a long time, and the vital forces of the economy show no indications of its toxic influence. But, when such quantity of the toxical principle has accumulated in the circulating fluid of the body as to produce pathological changes in the blood, or to directly spend its force upon the brain and spinal cord, its toxical effects are sure, speedy and powerful, and the victim is stricken down in an instant, unawares, when probably there had been no prodromic signs whatever, that might have been considered even a slight indisposition.

These opinions I have formed from observations made during the recent epidemic, of which I have already made mention; but my mind had previously been moved in that direction during former epidemics that have occurred under my immediate notice since 1848. That the poison, whatever it may be, or however it may be produced, is cumulative, there can be no doubt; because, during the prevalence of an epidemic of spotted fever, every person seems to be subject, to a greater or less degree, to manifestations of its influence, and the reason why one person is attacked and another escapes, is probably the peculiar susceptibility of the one, and the power of resisting morbid influences in the other.

During an epidemic of this disease, let any individual fall sick of any other disease, so that his vitality becomes impaired, and you will most certainly observe many of the symptoms of this disease manifested during the progress of the disease under which your patient is suffering. This holds good and is abundantly proven by the observations of every medical man who has passed through an epidemic of cholera or yellow fever; and there is no epidemic disease that is not more or less influenced by the type and character of the endemic diseases of the country where it may exist; and especially is this the case where our miasmatic fevers are most common. Probably no epidemic manifests this peculiarity more generally or more certainly than the disease under consideration, because many of its characteristic symptoms are inevitably present in all the endemic diseases that prevail at the time of the prevalence of the epidemic. I have treated very many children, and carried them successfully through an attack of infantile pneumonia to convalescence, and then, just when I was about to discharge the little sufferer as no longer needing medical care, and commenced congratulating the parents on the restoration of their darling to the blessings of health, this infernal disease—this so-called cerebro-spinal meningitis—would make its appearance with all its afflicting horrors, and, in spite of all the medical aid that could be rendered, it would carry its victim to the grave. That this disease has a malaria-miasmatic origin I have no doubt; but what the difference in the constitution of the poison consists in, that would designate it from that of our autumnal fevers, or from that of our typhoid fevers, is a question that is not yet solved, neither am I at this time able to answer. Nevertheless, it is true that neither malaria, proper, nor miasmata, has ever been made palpable by the most carefully directed experiments; neither have their existence been demonstrated by the most delicate chemical reagents. The spore theory may settle some of these difficulties in regard to intermittents, but that is far from receiving the unqualified sanction of the profession. Yet, that marsh miasm—whatever that may be—produces our intermittent and remittent fevers, there is no longer any doubt, and the presence of such poison is made evident from the circumstances that demonstrate its existence. So it is, too, with typhoid fever; it has its own special cause, because there is no other disease that runs the course that it does, or that manifests the same phenomena; neither are there any that produce the same pathological results. It was by the closest scrutiny and the most diligent observation of the circumstances and surroundings that attend the development of this disease, that has caused the profession to settle down in the conviction that it requires the decomposition of both animal and vegetable matter to produce that kind of poison, or malaria, that will favor the production of the latter disease; hence we see it most frequently making its appearance in crowded and illy ventilated apartments, where not only the animal exhalations from the body and lungs are present, but the excrements and other debris are not wanting to induce a ferment that is sufficient to poison the occupants of the premises.

In the vicinity of Spring Bay, and up and down the Illinois river, where cerebro-spinal meningitis, so-called, made its appearance by a most fearful and sudden mortality among those attacked during the fall and winter, the country for miles in every direction, excepting east, and even in front of the town, is a continued marshy swale, filled with lagoons; so that during the summer and autumn months, at least, the atmosphere cannot possibly be in any other condition than rife with marsh miasm; and what tends greatly to make the matter worse, the cattle, hogs and horses of the surrounding country, in seeking food, are constantly miring down and dying in these marshes, so that a combination of malaria and marsh miasm is, at one and the same time, generated and evolved, that is sufficient to poison the atmosphere for miles back into the country.

What other factor or entity may be present in connection with malario-miasmata, that forms a new and distinct product, has not so far been ascertained; but certainly a concatenation of circumstances, such as temperature, moisture, or electricity, or the whole combined, may be present, so that the three factors, or the two that are known, may be so acted upon by unknown agencies as to develop a third, the toxic action of which is greater than either one of its original constituents. This may be instanced by an illustration in chemistry, where the union of amygdalin and emulsin, either of which, separately, is innoxious, but united they form hydrocyanic acid, one of the most deadly poisons known, and which has no resemblance physically or therapeutically, to either of its constituents. It need not be said by the opponents of this theory, that the epidemic form of this disease always comes in the fall, winter and spring, and therefore there is no chance for decomposition and the consequent evolution of poison. Because, it is not the fact, according to the history of this disease, that it always makes its appearance in cold weather, for it has been known to appear as an epidemic in every month of the calendar.

The people of Woodford county seem to have had a mania for underground cellars, and nearly every respectable dwelling in the county has a cellar under it. These are stored full during the winter season of every kind of vegetables, and to keep them from freezing, every aperture is closed up, so that neither air, frost nor light can enter, making every one a dungeon, deep, dark and damp, without any available means of ventilation. These cellars are generally without means of drainage, on account of the lay of the land, and even where there is an opportunity for drainage, the nature of the soil and underlying clay precludes the possibility of their being kept dry, and, therefore, both winter and summer, they are damp and noisome, even when there is abundant opportunity to give them light and ventilation. During the winter season, on account of this moisture, heat and stagnant air, all the vegetables are undergoing the process of decay to a greater or less extent. In these noisome holes are also deposited the offal of meat and other animal remains; hence, the heat of the cellar during the winter months is capable of generating a malario-miasmata, the toxic influences of which must produce the most deleterious effects upon all who come within its range. This being undoubtedly true, it is a question worthy of further investigation, whether a poison generated under such circumstances, and at a temperature much of the time below 60° F., may not render its properties peculiar, and make it more inimical to human life than when produced under other and varied circumstances.

This disease-producing cause, whatever it may be, and however it may be produced, acts, through the blood, upon the brain and spinal cord, but, whether there is any material change produced in the constitution of the blood by the presence of the poison in that fluid before the manifestation of its toxical effects, is a point that is not yet settled, and certainly deserves further investigation; neither is it settled whether, or not, the poison is simply carried through the system by means of the blood, without change of either, to spend its force upon the great nerve-centres. All these things require further investigation, but now that we think we have reached the entrance of the bay that leads to a safe haven, we may confidently expect the explorations to continue till the lineal survey will be made complete, and the corner stones planted that will mark the boundary. But, that the poison does spend its force upon the great nerve-centres there is no longer any doubt; and that it is the primary cause of spotted fever, there can now be no question.

I will here make the statement, that the disease so produced is a distinct and entirely different disease from that of acute inflammation of the meninges of the brain and cord, or either of them, the latter of which is necessary in order to constitute a case of true cerebro-spinal meningitis. This proposition is not simply inferred; it is proven and made evident from numerous post mortem examinations that have been made by different observers in cases of sudden death from this disease; and in persons who have died prior to the sixth day there has never been found any appearance of inflammation, neither have any of its results been manifest; hence, individually, I have settled down in the conviction, that this is not, primarily, an inflammatory disease; and I am further convinced that any inflammatory action that may be developed during its progress is of a special or erysipelatous character, is secondary in its development, and never occurs till after the fifth day from the first decided manifestation of the disease. The action of the poison that produces this disease upon the nerve-centres, is the production of almost complete paralysis of their functions, and, hence, the delicate capillaries of the brain and cord, and their meninges, become, also, paralyzed and unable to carry on the circulation, in consequence of which, their nutrition is disturbed, and the physiological disintegration and molecular formation of new tissue is retarded, which leaves them in a pathological state. These capillary vessels, in consequence of the paralysis, become engorged with blood and pour out the watery contents of that fluid into the unyielding bony cavities, not in the form of an effusion indicative of resolution from an inflammation, but it is poured out by exudation through these paralyzed vessels, as through a dead membrane; and this not only produces pressure upon the cord itself, but the retained corpuscles and other constituents of the blood that are incapable of exudation tend to accumulate and engorge these atonic vessels, so that they, in conjunction with their exuded contents are capable of producing an amount of pressure upon the nerve-centres that, taken in connection with the poison, could scarcely be considered in any other light than making a death-stroke at their vital functions. This engorged condition of the atonic capillaries is, no doubt, what has given rise to the light pinky appearance of the membranes so often seen after a sudden death from this disease, and misled pathologists to that extent as to cause them to conceive that inflammation had been going on in the tunics of the brain and cord. The pressure that is produced by the exuded serum, assisted by the engorged vessels, is sufficient of itself to destroy the functions of the nerve-centres, independent of any damage that may have been done to the vital forces from the paralyzing influence of the toxic principle which was the immediate cause of the failure of their normal functions. This has been made apparent by all the post mortem examinations that have ever been made; because, in no instance has such examination ever developed any signs of inflammation, either in the brain or cord, or their investing membranes, where the victim has succumbed to the disease in a less time than six days from the attack; but, where the person has survived for from twelve to sixteen days, we then have signs of inflammation present, and such signs too as the disorganization of the great centres by softening, and pus between the layers of the arachnoid membrane and in the lateral ventricles. In private practice it is very difficult to get the consent of friends to a post mortem examination in any case, and in this disease, particularly, where the young are most frequently its victims, they think it seems like sacrilege to submit their dear departed ones to such examination. In army practice, however, the case is different, because it is there made a duty devolving upon hospital surgeons to make all investigations necessary in special cases for the general information of the medical staff, and report the same to the surgeon-general. During the months of March and April, 1862, while I had the honor of having charge of the general hospital at Smithland, Ky., there occurred many cases of this disease among the invalid Federal soldiers who were left behind, in camp, previous to the battles of Shiloh and Donaldson. Many of those who were attacked died, and as the opportunity was offered I selected the subjects as they occurred, making two examinations of men who died previous to the sixth day, and two of persons who survived till after the twelfth day. The two who died within six days from the time of attack were found to have an unusual amount of fluid in the cavities, and the membranes were of a light pink color, but there was no flaky appearance of the liquid, nor was there any semi-organized membranes in connection with the tissues. The substance of the brain and cord, so far as the unaided eye could distinguish, was, apparently, in a healthy condition. The two, however, who lived to near the sixteenth day showed a very different condition of the substance of the brain and cord. These two cases, about the seventh day began to be feverish and more restless, and the temperature in the rectum showed 103° F., whereas previously the temperature had not reached 100° F.; their pulse became more frequent, and they had some muttering delirium; neither of them became violent, but remained much in the same condition, their pulse becoming more frequent and feeble as the end approached. One of these patients had convulsions towards the last, and the other had paralysis of one side. The autopsia cadaverica in the one having convulsions showed a sero-sanguineous fluid at the base of the brain and within the spinal canal, and when the cord was lifted from its bed, portions of it dropped off, and the membranes were unusually tender. In the case of the one having paralysis there was slight softening of the Pons varolii, bloody serum at the base of the brain and in the spinal canal, and also in the lateral ventricles—this patient was blind, and the pupils were dilated and insensible to light for three or four days previous to his death.

I am stating these facts from memory, my memorandum having been lost or mislaid before I left the service. These two latter cases were both evidently subjects of a special inflammation which was more allied to that of erysipelatous than that of acute or any other character of inflammation; and this was never set up till on or after the sixth day from the attack, as was evident from the thermometrical range of temperature after that period; and in both instances it would appear that not only the enveloping membranes, but also the substance of the brain and cord, were attacked with this special inflammation, which went speedily on to disorganization.

And now, independent of these illustrations, it is a conceded fact, deduced from the most careful and accurate observation, that in no epidemic of this disease (so-called cerebro-spinal meningitis) have the reputed antiphlogistic remedies been of any avail; in fact, when they have been resorted to, with the idea of allaying acute inflammation, they have proven worse than useless, and been a direct injury to the patient wherever and whenever they have been resorted to in the treatment of this disease.

With regard to the treatment that we have found most satisfactory in this disease, I will here make the statement, that since 1863, my brother, Z. H. Whitmire, and I, have treated this so-called cerebro-spinal meningitis in accordance with the pathology herein laid down, and that too with the most satisfactory results; more satisfactory, indeed, than any that have been obtained from any other given method. In saying this, I can assure you that we have no routine practice to offer you, nor specifics to recommend, because no two cases are precisely alike; hence the indications to be met in each case will vary in minor particulars, but we always endeavor to keep steadily in view one prominent feature of this disease, and that is, the immediate and fearful tendency to the failure of the vital functions which is apparent in the beginning of every sudden and violent attack. This low status of the vital forces is not unfrequently manifested by a morbidly natural, and occasionally a slow soft pulse, a cool pallid surface, and a general apathy of the patient's movements. This condition is sometimes succeeded by a feeble attempt at reaction and restoration of the vital functions; when, instead of a perfect restoration, restlessness will supervene, and then, instead of the slow measured beat of the pulse that was present at the outset of the disease, it will become frequent and unsteady, but it will still lack the force that an acute inflammation would produce, and the patient may or may not fall into a low muttering delirium. He will never, however, at this stage, be thrown into that wild ungovernable mania that is wont to follow an acute inflammation of the meninges of the brain and cord. A wild delirium, however, may supervene, either on or after the sixth day, and when it does, it is an unqualified indication of the commencement, setting up and progress of that special inflammation of which I have previously made mention in this paper.

The indications of treatment, therefore, in the commencement of an attack, are to be met with evacuants, eliminants, stimulants, anodynes, tonics, rubefacients, and the hot bath or pack, in connection with the most nutritious food from the commencement, that is capable of being assimilated by the patient. We use evacuants because as a rule the bowels are in a constipated condition, but it is best to use them only to that extent which is sufficient to once thoroughly cleanse the alimentary canal; and that being done, be sparing in the use of purgatives in the after treatment. We may expect favorable results, also, from the use of eliminants on account of their supposed action in facilitating the removal of morbific material from the economy through the emunctories—skin, liver, kidneys and lungs. Stimulants are necessary that the vital forces may be sustained, and thus render aid to the action of the eliminants and enable them to do their work without too great a draft upon the vitality of the system. Anodynes may be used, if necessary to produce rest and sleep, for in that condition there are greater and more successful efforts at recuperation than in any other condition of the system, the value of which, in this regard, would be a work of supererogation to present to the members of these societies. We would use tonics for the same reasons that would lead us to use stimulants; their action is slower, but their ultimate effects are more permanent and the force of the heart's action needs to be sustained. Rubefacients are specially useful in this disease, and more particularly the tincture of iodine, which, besides its powerful revulsive effects, by its absorption from the surface acts as a tonic to paralyzed capillaries and excites the absorbents to action, and may thereby relieve the cord from pressure by the taking up of any fluids that may have been poured out of the vessels, and cause the engorged capillaries to empty themselves into the general circulation, and thus, probably avert the secondary effect of the poison, which is the supervention of a special or erysipelatous inflammation of the brain and cord or their investing membranes.

In case we should be called to see a patient and find him languid, surface cool or natural, pulse 60 to 70, occasional wandering, or not, as the case may be, constipation of the bowels, an intense pain in the back of the head, neck and along the dorsal spine, and complaining of a stricture about the chest, so as to prevent a deep inspiration, and which causes frequent sighing; we at once order him enveloped in a wet blanket wrung out of *hot* water, and apply bottles of hot water along the spine and to the extremities, or, what is still better and always at hand, we put into a boiler, with water, half a bushel of ear-corn and bring it to the boiling point; we then pile the hot ears along the spine from the nape of the neck to the hips; and also distribute the corn along and about the extremities, and cover both the patient and corn with one or two thick cotton comforters. In this pack we keep our patient from thirty minutes to one hour, occasionally changing the corn so as to keep it *hot* till he is in a profuse perspiration. The patient will not be in the pack more than thirty minutes till he will drop into a quiet sleep, and so we let him rest, sometimes for an hour, quietly changing the corn as it loses its heat, for other ears of a higher temperature, so as to prevent the pack from becoming simply a warm bath. After a reasonable time we take him out of the pack, give him a thorough rubbing with a dry coarse towel and put him into a dry, warm bed; and as often as our patient becomes restless, or complains of great pain, we repeat the pack —not a warm pack—because we consider that the stimulation of the *hot* pack is one of the essentials necessary to meet the indications in the case by relieving the nerve-centers of their engorgement and enabling the functions of animal life to be carried on more perfectly. It quiets restlessness, allays pain, and produces tranquil sleep. The *hot* pack we have often resorted to, three, four and five times during twenty-four hours, to accomplish these objects, and never without producing the most happy effect. It has never disappointed us in its results, and hence we have never had occasion to regret its timely and diligent application. We are not unaware that, under these circumstances, the historical ice-bag has been resorted to with the idea of reducing or preventing inflammatory action, but in our hands it has never appeared of any practical value; on the contrary, its action has rather proven mischievous than otherwise until after the sixth or seventh day, when it is more than probable that a special inflammatory action has taken place in the brain and cord, or their membranes, or both. After the *hot* pack, we usually advise the administration of a mild cathartic, for the purpose of removing any acrid or other secretions that might prove a source of irritation and disturbance during the course of the disease.

The alimentary canal once cleansed, we then put our patient under constitutional treatment, and for an adult, or during the latter term of adolescence, our average prescription would be: R. Pot. brom., dr. j.; gelsem. fluid ext., dr. j.; ol. terebinth, dr. j.; aqua, oz. j.; syrup simpl. oz. iij. The dose of this mixture is one tablespoonful every four hours. As a stimulant and tonic, to give force and permanency to the heart's action, to aid or facilitate the oxidation of the blood, and to improve the secretions so as to favor the elimination of the poison, we have found the following recipe admirably adapted for that purpose: R. Opii pulv., grs. iv.; ipec. pulv., grs. vi.; quin. sulph., sc. j.; ammon. mur., pot. chlor., *aa* dr. j. Mix and divide into twelve powders, one of which may be given between each dose of the mixture. We also advise the muriated tinct. of iron, to be given in twenty drop doses three times a day from the commencement of the treatment. This would be simply an average prescription for an adult; for children it should be proportional, but the manifest object of the prescription in either case should not be lost sight of, and the intelligent practitioner will often see good and sufficient reasons for many minor changes or additions to what we have here recommended in almost every case he meets. Many cases have come under our care where we have been compelled to resort to the hyd. of chloral for the purpose of inducing sleep, and so far we have never seen any evil consequences resulting from its use. The physiological or therapeutic action of each of the several medicines, herein recommended, is too well known to the profession for me to trench upon your valuable time in a dissertation upon their separate or combined action upon the economy; but it may be well in this place to mention that the acknowledged action of bromide of potassium is specially directed to the brain and spinal cord, and through a reflex influence produced by the drug, the calibre of the capillaries of the nerve-centres is lessened, thus enabling them to disgorge themselves, and probably, like the iodide of potassium, it also stimulates the paralyzed absorbent vessels into renewed activity, so that the supernatent fluid in the cavities is taken up, thus relieving the brain and cord of the compression to which they have been subjected by their presence. It may not be amiss in this place to also mention that gelseminum is comparatively a new remedy with the regular profession, and its complete physiological action upon the economy is not, probably, fully known; but, in these cases, it seems admirably adapted to allay pain, to compel quiet where there is great restlessness, and to insure sleep; besides, so far as we have observed, while it may lessen in some degree the frequency of the heart's action, it does not in the least abate its force; so that we may safely conclude that the frequency of the heart's action is only abated on account of the tranquility that the drug is wont to produce, and not on account of any direct sedative influence produced upon the nerve-centres and through them influencing the action of the heart and arteries. Therefore we have reckoned this drug as one among our most efficient remedies in this disease; one, too, that, in our opinion, cannot well be dispensed with; because it not only exercises a controlling influence over the cerebro-spinal system, but it induces diuresis and diaphoresis at the same time—a quality that is rarely, if ever, found in any other drug. As a rubefacient, we prefer, by all odds, the tinct. of iodine, which is applied along and on each side of the spinal column twice a day until the skin becomes tender, when we mix it with castor oil, to render its action milder and avoid vesication. From the known action of iodine we expect it to operate as an adjuvant to the bromide of potassium in setting the lymphatic vessels into such activity that the accumulated fluid in the unyielding cavities will speedily be taken up, the pressure removed from the brain and cord, and the vital forces permitted once more to resume their sway.

Finally, Mr. President, and gentlemen, I am exceedingly gratified to be able, here, and at this time, to make the announcement, with becoming modesty, and without any spirit of boasting, that since 1863, we have passed through three most terrible epidemics of this disease within our range of practice; and under this general plan of treatment, my brother and I have not lost to exceed fifteen per cent. of our patients who were afflicted with spotted fever, which we think is a sufficient guarantee to induce us to continue in well-doing, and to recommend this subject to the careful consideration of our professional brethren.

